# Diagnosis of patent ductus arteriosus by different echocardiographic methods in very preterm infants

**DOI:** 10.1007/s00431-025-06485-y

**Published:** 2025-09-26

**Authors:** Carlo Dani, Davide Sarcina, Iuri Corsini, Simone Pratesi, Chiara Poggi, Simona Montano, Barbara Loi, Giulia Regiroli, Daniele De Luca

**Affiliations:** 1 Department of Neurosciences, Psychology, Drug Research and Child Health, University of Florence, Florence, Italy; 2 Division of Neonatology, Careggi University Hospital of Florence, Largo Brambilla 3, Florence, 50134 Italy; 3 Division of Pediatrics and Neonatal Critical Care, A. Béclère Medical Center, Paris Saclay University Hospitals, Paris, France

**Keywords:** Patent ductus arteriosus, Diagnosis, Echocardiography, Preterm infant

## Abstract

There is no consensus regarding the timing and diagnostic criteria for identifying hemodynamically significant patent ductus arteriosus (hsPDA). Our aim was to evaluate if the use of different diagnostic criteria at different times could be associated with a different incidence of hsPDA in very preterm infants. We studied 41 infants with gestational age < 32 weeks born in neonatal intensive care units (NICU) in Florence, Italy, or in Paris, France. They received the first echocardiography between 24 and 48 h of life and the second between 72 and 84 h to diagnose hsPDA using Florence and Paris criteria and PDA severity score. Concordance of diagnosis between criteria was evaluated with the Cohen unweighted κ statistic. The incidence of hsPDA diagnosed by the Florence (35%) or Paris (34%) criteria or by PDA severity score (35%) was similar. Concordance was substantial between Florence and Paris criteria and between Florence criteria and PDA severity score but was fair between Paris criteria and PDA severity score. Moreover, concordance significantly changed from the first to the second echocardiography.

*Conclusion*: The studied diagnostic criteria showed important variations of concordance when applied at different times. This led to diagnose hsPDA in different patients at different times while leaving the overall percentage of hsPDA unchanged. Our results suggest that more attention should be paid to the choice of diagnostic criteria for individuating hsPDA in very preterm infants.

**Table:** 

**What is Known:** *• There is no consensus regarding the timing and diagnostic criteria for individuating hemodynamically significant patent ductus arteriosus (hsPDA) in preterm infant.* *• This implies large variations in its frequency and management diagnosis between centers.* **What is New:** *• This study evaluated for the first time how different diagnostic criteria used at different postnatal times may influence the diagnosis of hsPDA in very preterm infants.*

**What is Known:**

*• There is no consensus regarding the timing and diagnostic criteria for individuating hemodynamically significant patent ductus arteriosus (hsPDA) in preterm infant.*

*• This implies large variations in its frequency and management diagnosis between centers.*

**What is New:**

*• This study evaluated for the first time how different diagnostic criteria used at different postnatal times may influence the diagnosis of hsPDA in very preterm infants.*

## Introduction

Patent ductus arteriosus (PDA) is a frequent complication in preterm infants with respiratory distress syndrome (RDS). Approximately 10% of infants born between 30 and 37 weeks of gestation have a patent ductus at 4 days after birth, but the frequency increases to 80% in those born between 25 and 28 weeks and to over 90% in those born at 24 weeks [1]. The Vermont Oxford Network reported that in 2024, 31% of infants born at less than 30 weeks’ gestational age received pharmacological treatment for PDA, and 3% underwent surgical closure [2]. How PDA should be managed is a subject of lively debate because randomized controlled trials of PDA closure using non-steroidal anti-inflammatory drugs failed to demonstrate significant benefits in preterm infants [3] often due to study design limitations [4, 5]. However, a persistent left-to-right shunt through the ductus arteriosus (DA) complicating RDS has been associated with a worsening of respiratory failure, lowering of the survival rate, increased risk of bronchopulmonary dysplasia (BPD), pulmonary hypertension associated with BPD, and impairment of neurodevelopment [6–9]. 

The current treatment of PDA consists of two steps: the first is pharmacological treatment and the second, if medical treatment fails, is surgical or trans-catheter closure, which should be avoided, if possible, due to the associated possible complications [10]. Since PDA diameter alone demonstrated weak correlations with the magnitude of shunt volume through the ductus [11], combinations of clinical and echocardiographic parameters have been proposed to identify hemodynamically significant PDA (hsPDA) that warrant treatment [11–13]. Brissaud et al. reported that 72% of 57 French neonatal intensive care units (NICU) used clinical and echocardiographic criteria to decide when to initiate PDA treatment, whereas 27.6% used only echocardiographic criteria, regardless of a patient’s clinical status. Moreover, they detailed that more than nine echocardiographic criteria were used for the diagnosis of hsPDA with different combinations [12].

Consistently, there is no consensus regarding the timing and diagnostic criteria for individuating hsPDA, and its assessment remains a challenge. This implies that there are large variations in the management of PDA [14] and large differences in the frequency (20–60%) of hsPDA diagnosis between NICUs [15].

### Aim of the study

Based on these considerations, we hypothesized that the use of different diagnostic criteria at different times could be associated with a different incidence of hsPDA in very preterm infants. To evaluate this hypothesis, we measured changes in the incidence of hsPDA observed using different diagnostic echocardiographic criteria (Paris and Florence criteria) and El-Khuffash’s PDA severity score [11, 16, 17] in a cohort of very preterm infants at two different European hospitals.

## Material and methods

### Patients

The study was carried out at the third level neonatal intensive care units (NICUs) of Careggi University Hospital, Florence, Italy, and A. Béclère Medical Center, Paris, France, after the approval of local ethics committees. Infants were included in the study after informed parental consent if they were born with gestational age < 32 weeks of gestation and admitted from February to August 2024. Exclusion criteria were major congenital malformations, chromosomal disorders, inherited metabolic diseases, and death in the first 72 h of life.

### hsPDA diagnosis and treatment

All patients underwent PDA assessment with the Florence criteria, the Paris criteria, and the PDA severity score. The diagnosis of hsPDA requiring pharmacological treatment was made in Florence by echocardiographic demonstration of a ductal left-to-right shunt, with a ductal size ≥ 1.4 mm/kg and two or more other echocardiographic criteria, while in Paris, it was made by the combination of two echocardiographic criteria with gestational age (Table 1). As per local protocol, the first echocardiography was performed between 24 and 48 h of life in Florence and between 72 and 84 h in Paris. Therefore, patients born in Paris performed the first heart ultrasound between 24 and 48 h of life to evaluate the results of the Florence criteria application and the second between 72 and 84 h of life to evaluate the results of the Paris criteria. Differently, patients born in Florence performed the second ultrasound only when the first one excluded the diagnosis of hsPDA and the need for treatment.

**Table 1:** Diagnostic criteria for hsPDA used in Careggi University Hospital, Florence, Italy, and A. Béclère Medical Center, Paris, France

Florence criteria	Paris criteria	
Infants born < 32 weeks of gestational age	Infants born < 28 weeks of gestational age	Infants born 29-31 weeks of gestational age
Ductal size ≥ 1.4 mm/kg and ≥ 2 of the following criteria: • left atrium to aortic root (LA/Ao) ratio > 1.5 • pulsatile flow pattern • reverse flow in descending aorta or superior mesenteric artery • left ventricular output > 300 ml/kg/min	> 2 of the following criteria: • ductal size ≥ 1.5 mm/kg or > diameter of LPA • atrium to aortic root (LA/Ao) ratio > 1.5 • resistance index > 0.9 in pericallosal artery • diastolic flow in LPA > 0.2 cm/sec	> 2 of the following criteria: • ductal size ≥ 1.5 mm/kg or > diameter of LPA • atrium to aortic root (LA/Ao) ratio > 1.5 • resistance index > 0.9 in pericallosal artery • diastolic flow in LPA > 0.2 cm/sec and ≥ 1 of the following clinical characteristics: • oxygen-dependency at 72 h of life • increase in systemic differential pressure concomitant with decrease in systemic pressure

*LPA* left pulmonary artery

The El-Khuffash’s PDA severity score was calculated immediately after echocardiographies using the formula: (gestation in weeks ×  − 1.304) + (PDA diameter in mm × 0.781) + (Left ventricular output in ml/kg/min × 0.008) + (maximum PDA velocity in m/s x − 1.065) + (Left ventricular a′ wave in cm/s x − 0.470) + 41 [11, 16, 17].

Echocardiographies were repeated at least at the end of each pharmacological course. All ultrasound studies were performed by pediatric cardiologists or by neonatologists trained for neonatal echocardiography (4 in Florence and 13 in Paris).

Infants who developed hsPDA were treated as per local protocol. Intravenous ibuprofen was given at the standard dose of 10 mg/kg followed, after 24 and 48 h, by 5 mg/kg. Intravenous paracetamol was given at a dose of 15 mg/kg/6 h for three days. Paracetamol was given as a first choice when patients had contraindications to ibuprofen (serum creatinine concentration > 1.5 mg/dL, urine output < 1 mL/kg/h or < 0.5 mL/kg/h during the first 24 h of life; platelet count < 50,000/mm^3^, IVH ≥ grade 3). High-dose ibuprofen, paracetamol, or the combination of ibuprofen plus paracetamol could be given when the first course of ibuprofen failed. The treatment was decided by the neonatologist on duty.

### Recording of clinical data

Clinical and demographic data were prospectively collected and reported in Table 2. BPD was defined as oxygen requirement at 36 weeks PMA [18]. NEC was defined as Bell’s stage 2 or higher [19]. IVH was diagnosed and staged according to the classification of Papile et al. [20]. ROP was diagnosed according to International Classification of ROP [21].

**Table 2:** Baseline clinical characteristics of the infants and their mothers whose PDA was evaluated for hemodynamic significance. Mean ± (SD), or rate and (%), or median and (IQR)

Characteristics	*N* = 41
Infants	
Gestational age (wks)	28.0 ± 1.9
Birth weight (g) < 10th percentile < 26 weeks of gestational age	986 ± 270 8 (20) 6 (15)
Female	20 (49)
Apgar score 5°min	8 (6–9)
Noninvasive ventilation	41 (100)
Mechanical ventilation	9 (22)
Surfactant	23 (56)
Pulmonary hemorrhage	6 (15)
Necrotizing enterocolitis	2 (5)
Bronchopulmonary dysplasia	15 (37)
Intraventricular hemorrhage	6 (15)
Death	5 (12)
Duration of hospital stay (d)	47 ± 22
Characteristics	***N*** = 41
Mothers	
Antenatal steroids	35 (85)
Cesarean section	29 (71)
Gestational diabetes	4 (10)
Hypertensive disorders of pregnancy	10 (24)
Chorioamnionitis	4 (10)

For each infant studied, we reported in Table 3 the diagnosis of hsPDA, measured echocardiographic parameters, treatment with ibuprofen and/or paracetamol, number of pharmacological courses, therapeutic success or failure, and need for surgical closure.

**Table 3:** Clinical characteristics, echocardiography parameters at the first and second echocardiography, and treatment of hsPDA in studied infants. Mean ± (SD), or rate and (%)

Characteristics	First echocardiography (*n* = 41)	Second echocardiography (*n* = 24)	*P*
Gestational age (wks)	28.1 ± 1.9	28.4 ± 1.9	0.541
Weight (g)	957 ± 255	896 ± 220	0.332
Noninvasive ventilation	33 (80)	12 (50)	0.022
Mechanical ventilation	9 (22)	12 (50)	0.040
Mean airway pressure (cm H_2_O)	8.0 ± 2.4	8.5 ± 3.3	0.484
SpO_2_/FiO_2_	435 ± 51	423 ± 62	0.401
Systemic systolic pressure (mmHg) Systemic diastolic pressure Mean systemic pressure	58 ± 9 37 ± 9 44 ± 8	58 ± 10 35 ± 10 41 ± 13	1.000 0.410 0.253
La/Ao ratio PDA diameter (mm) PDA diameter (mm/kg)	1.3 ± 0.2 1.4 ± 0.5 1.4 ± 0.7	1.5 ± 0.2 1.6 ± 0.4 1.7 ± 0.7	< 0.001 0.010 0.100
Peak velocity of PDA (cm/sec)	1.9 ± 0.5	1.8 ± 0.8	0.537
Pulsatile flow pattern	13 (32)	12 (50)	0.231
Left ventricular output (ml/kg/min)	206 ± 67	278 ± 81	< 0.001
Left ventricular a′ wave (cm/sec)	5.5 ± 1.8	4.5 ± 0.9	0.014
Diastolic flow in LPA (cm/sec)	0.15 ± 0.10	0.29 ± 0.28	0.005
Absence of arterial mesenteric diastolic flow	5 (12)	11 (46)	0.006
Cerebral resistant index	0.73 ± 0.06	0.79 ± 0.09	< 0.001
PDA severity score	2.9 ± 2.7	4.8 ± 2.6	0.007
Pharmacological treatment of PDA	19 (46)		
One course of ibuprofen Two courses of ibuprofen	15 (37) 5 (12)		
One course of paracetamol Two courses of paracetamol	8 (20) 5 (12)		
Ibuprofen plus paracetamol	4 (10)		
Surgical closure	1 (2)		

*LPA* left pulmonary artery

### Statistical analysis

The quantitative clinical characteristics of infants were described as mean and SD for normally distributed values or as median and IQR for nonnormally distributed variables. Categorical variables were reported with frequencies and percentage. Continuous and categorical variables were compared using the Student *t* test or the Wilcoxon rank sum test for non-parametric continuous variables and Χ^2^ test for categorical variables, respectively. A P value < 0.05 was considered statistically significant.

Concordance between Florence criteria, Paris criteria, and PDA severity score for the diagnosis of hsPDA was evaluated with the Cohen unweighted κ statistic. Kappa statistics was interpreted as following: less than 0.2 represents poor agreement; 0.2–0.4 represents fair agreement; 0.41–0.6 represents moderate agreement; 0.61–0.8 represents substantial agreement; greater than 0.8 represents great agreement [22].

Assuming a *κ* coefficient of correlation of 0.85 between Florence and Paris echocardiographic criteria, a sample size of 65 echocardiographies was calculated to obtain a statistical power of 90% with *α* = 0.05.

## Results

We studied 41 infants (8 in Florence and 33 in Paris) with a gestational age of 28.0 ± 1.9 weeks and a birth weight of 986 ± 270 g whose clinical characteristics are detailed in Table 2. Patients underwent the first (*n* = 41) and second (*n* = 24) echocardiography at 28.1 ± 1.9 and 28.4 ± 1.9 weeks of gestational age, respectively.

We observed that La/Ao ratio, left ventricular cardiac output, diastolic flow in LPA, absence of arterial mesenteric diastolic flow, cerebral resistant index, and PDA score increased from the first to the second echocardiography, while left ventricular a′ wave decreased (Table 3).

The overall incidence of hsPDA diagnosed by the Florence (35%) or Paris (34%) criteria or by PDA severity score (35%) was similar. The frequency of hsPDA increased from the first to the second echocardiography, but the difference was statistically significant only for the Florence and Paris criteria (Table 4).

**Table 4:** Frequency of hsPDA using Florence criteria, Paris criteria, or PDA score. Rate and (%)

	All echocardiographies (*n* = 65)	First echocardiography (*n* = 41)	Second echocardiography (*n* = 24)	*P* 1 st vs 2nd echocardiography
Florence criteria	23 (35)	8 (20)	15 (63)	< 0.001
Paris criteria	22 (34)	5 (12)	17 (71)	< 0.001
PDA score	23 (35)	11 (27)	12 (50)	0.06

Nineteen (46%) infants had pharmacological treatment for hsPDA, 12 (29%) in Paris and 7 (17%) in Florence. Among them, 15 (37%) were treated with ibuprofen and 8 (20%) with paracetamol. Four (10%) patients were given both ibuprofen and paracetamol (Table 3). Among untreated infants, only one patient had a diagnosis of hsPDA (with PDA severity score). Surgical closure of the PDA was necessary in only one patient.

**Fig. 1:**
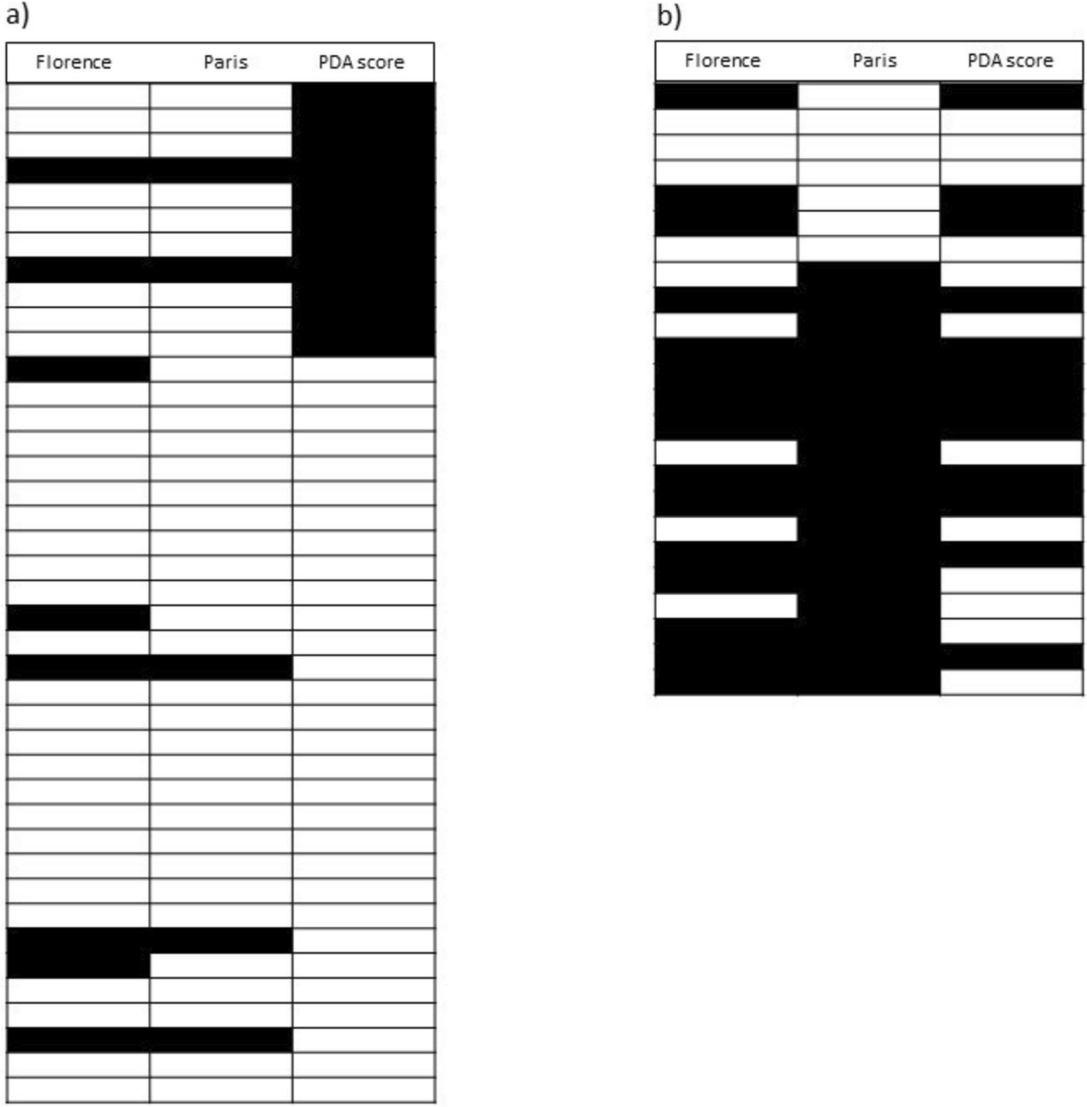
Diagnostic results of Florence criteria, Paris criteria, and PDA severity score at **a** the first and **b** the second echocardiography. Each white block represents a diagnosis of no hsPDA, while each black block represents a hsPDA diagnosis

The Florence and Paris criteria had an overall concordance of 83% (54/65 diagnoses), with a κ statistic of 0.626 (95% C.l. 0.427–0.826). The Florence criteria compared with the PDA severity score had an overall concordance of 85% (55/65 diagnoses), with a κ statistic of 0.664 (95% C.l. 0.473–0.854). The Paris criteria compared with the PDA severity score had an overall concordance of 64% (42/65 diagnoses), with a κ statistic of 0.219 (95% C.l. −0.026–0.463). At the first echocardiography, Florence criteria had a moderate agreement with Paris criteria and PDA severity score, while Paris criteria had a fair agreement with the PDA severity score. At the second echocardiography, Florence criteria had a fair agreement with Paris criteria and moderate agreement with the PDA severity score, while Paris criteria had a poor agreement with PDA severity score (Table 5, Fig. 1).

**Table 5:** Concordance and *k* statistic between Florence criteria, Paris criteria, and PDA score for the diagnosis of hsPDA. Rate and (%) or *k* statistic and (95% CI)

	All echocardiographies (*n* = 65)	First echocardiography (*n* = 41)	Second echocardiography (*n* = 24)
Florence vs Paris criteria Concordance *k* statistic	54 (83) 0.626 (0.427–0.826)	38 (93) 0.728 (0.444–1.000)	16 (67) 0.256 (− 0.140–0.652)
Florence criteria vs PDA score Concordance *k* statistic	55 (85) 0.664 (0.473–0.854)	34 (83) 0.524 (0.220–0.828)	21 (88) 0.750 (0.494–1.000)
Paris criteria vs PDA score Concordance *k* statistic	42 (64) 0.219 (− 0.026–0.463)	29 (80) 0.399 (0.081–0.718)	13 (54) 0.083 (− 0.279–0.446)

## Discussion

In this study, we evaluated for the first time how different diagnostic criteria used at different postnatal times may influence the diagnosis of hsPDA in very preterm infants. We found that the Florence and Paris criteria and the PDA severity score detected a similar rate of hsPDA diagnosis. Furthermore, subanalysis of the results showed that the first echocardiography detected a lower frequency of hsPDA than the second, with no differences among the three diagnostic criteria. This finding can be explained by the increase of values of most echocardiographic parameters included in the Florence and Paris criteria and in the PDA severity score. In fact, PDA diameter, left atrium to aortic root ratio (LA/Ao), left ventricular output, diastolic flow in left pulmonary artery (LPA), cerebral resistance index, and the absence of arterial mesenteric diastolic flow were increased in the second echocardiography.

However, when we calculated the concordance between the three criteria, we found significant diagnostic differences. This suggests that choosing one criterion rather than another could lead to treating different patients at different times while leaving their overall percentages unchanged. Overall, the Florence and Paris criteria showed a substantial agreement, but this was due to the results of the first echocardiography and not of the second, since their concordance were 93 and 67%, respectively. The Florence and PDA severity score criteria showed a substantial agreement, but the agreement was moderate (83%) at the first echocardiography and substantial (88%) at the second. Finally, the Paris and PDA severity score criteria had a fair agreement since the concordance was fair (80%) at the first echocardiography and became poor (54%) at the second.

These differences were likely due to the difference between the criteria studied, pathophysiological changes over time in the measured ultrasound parameters and, probably to a lesser extent, to the clinical parameters included in the Paris criteria and the PDA severity score [11, 12]. In particular, the criteria for diagnosing hsPDA are likely to be influenced by the decrease in pulmonary vascular resistance and the increase in right and left ventricular output that are expected to occur during the transition phase [23, 24]. In any case, these results clearly demonstrated that the choice of the diagnostic criteria and the timing of their application can influence hsPDA diagnosis and treatment.

Considering these findings, it is reasonable to ask whether there are better parameters or scores than those we studied for diagnosing hsPDA, such as the IOWA PDA score [25]. However, it is not possible to provide an answer at this time, given the complexity of the condition, the large number of parameters measurable at different postnatal ages, and the subjectivity of the measurements.

It can be observed that the frequency of hsPDA in our population was quite high. We believe this is due to the short duration of enrollment, which caused a selection bias, and that the high frequency of hsPDA was due to chance.

Our study has some limitations. The two participating centers used local criteria for the diagnosis of hsPDA and to decide its management, and neither of them used the PDA severity score. Therefore, we could not effectively evaluate the effects of applying these diagnostic methods on the management of PDA in clinical practice, as a randomized controlled trial could do. In addition, we achieved the planned sample size of 65 total echocardiographies, but the number of the first and second echocardiographies was lower, and the corresponding subanalyses may not have optimal statistical power. The study population was < 32 weeks of gestation and, therefore, our results may not reflect the conditions of more immature infants, which limits the generalizability of the findings.

In conclusion, the studied diagnostic criteria showed important variations of concordance when applied at different times. This led to diagnose hsPDA in different patients at different times while leaving the overall percentage of hsPDA unchanged. Our results suggest that more attention should be paid to the choice of diagnostic criteria for individuating hsPDA in very preterm infants.

## Data Availability

Data are available on reasoned request.

## Abbreviations

BPD: Bronchopulmonary dysplasia
hsPDA: Hemodynamically significant patent ductus arteriosus
NICU: Neonatal intensive care units
PDA: Patent ductus arteriosus
RDS: Respiratory distress syndrome
